# Quantitative trait loci mapping for canine hip dysplasia and its related traits in UK Labrador Retrievers

**DOI:** 10.1186/1471-2164-15-833

**Published:** 2014-10-01

**Authors:** Enrique Sánchez-Molano, John A Woolliams, Ricardo Pong-Wong, Dylan N Clements, Sarah C Blott, Pamela Wiener

**Affiliations:** The Roslin Institute and Royal (Dick) School of Veterinary Studies, University of Edinburgh, Easter Bush, Midlothian, EH25 9RG Scotland, UK; Royal (Dick) School of Veterinary Studies, University of Edinburgh, Easter Bush, Midlothian, EH25 9RG Scotland, UK; Kennel Club Genetics Centre at the Animal Health Trust, Lanwades Park, Kentford, Newmarket, Suffolk, CB8 7UU UK; School of Veterinary Medicine and Science, University of Nottingham, Sutton Bonington, Leicestershire, LE12 5RD UK

**Keywords:** Canine hip dysplasia, Labrador Retriever, Norberg Angle, QTL, GWAS, Genomic selection

## Abstract

**Background:**

Canine hip dysplasia (CHD) is characterised by a malformation of the hip joint, leading to osteoarthritis and lameness. Current breeding schemes against CHD have resulted in measurable but moderate responses. The application of marker-assisted selection, incorporating specific markers associated with the disease, or genomic selection, incorporating genome-wide markers, has the potential to dramatically improve results of breeding schemes. Our aims were to identify regions associated with hip dysplasia or its related traits using genome and chromosome-wide analysis, study the linkage disequilibrium (LD) in these regions and provide plausible gene candidates. This study is focused on the UK Labrador Retriever population, which has a high prevalence of the disease and participates in a recording program led by the British Veterinary Association (BVA) and The Kennel Club (KC).

**Results:**

Two genome-wide and several chromosome-wide QTLs affecting CHD and its related traits were identified, indicating regions related to hip dysplasia.

**Conclusion:**

Consistent with previous studies, the genetic architecture of CHD appears to be based on many genes with small or moderate effect, suggesting that genomic selection rather than marker-assisted selection may be an appropriate strategy for reducing this disease.

**Electronic supplementary material:**

The online version of this article (doi:10.1186/1471-2164-15-833) contains supplementary material, which is available to authorized users.

## Background

Canine hip dysplasia (CHD) is a pathology characterised by the malformation of the coxo-femoral joint, leading to degeneration of the hip joint, lameness and painful osteoarthritis. It mainly affects large-sized breeds like the German Shepherd and Labrador Retriever and has an important impact due to its high prevalence in these breeds (25-40% in UK Labrador Retrievers [1], depending on the study). The disorder is a major health concern of dog owners, breeders and organizations (e.g. the American Kennel Club [2]) and cannot be cured, although it can be ameliorated by surgery and there is some suggestion that modification of certain factors (e.g. reduction of dietary calcium and vitamin D in growing dogs, [3]) may improve the condition. Although surgical/medical treatment can be used to improve the quality of life of the dog, it has been shown that CHD has a moderately heritable genetic basis [4] and due to the difficulty of treating the disease, a genetic solution should be explored.

A genetic strategy relies on selecting breeding individuals on the basis of their predicted breeding value, with greater accuracy of prediction offering faster progress. To date these predictions have relied on mass selection on a phenotype measured at a young age, and often prior to the onset of clinical disease. In many countries, this phenotype is based on a radiographic analysis of the pelvic area, and in the UK this scheme is run jointly by the British Veterinary Association (BVA) and The Kennel Club (KC) [5]. In this scheme, animals older than one year of age are scanned, when skeletal maturity is assumed to have been achieved (although no upper age limit is imposed), and from the radiograph, hip score (HS) is calculated as the sum of nine component traits measured on categorical scales on both hips. Thus, hips with a perfect radiographic appearance will have a HS of 0, and the higher the score (up to 106), the greater the degree of CHD and/or degenerative change. Three of these component traits (Norberg Angle, Subluxation and Cranial Acetabular Edge) are measures of joint laxity. The other six are related to osteoarthritis which develops as a result of joint laxity, and therefore are more subject to detrimental age effects [6]. Lewis *et al*. [6] also observed that the measures of laxity were more heritable than those associated with osteoarthritis.

Current breeding programmes against the disease are voluntary and recommend breeding from animals with HS (or equivalent) below a given threshold. They have resulted in measurable but moderate success [7–10]. Approaches that could enhance the performance of these programmes include selection based on phenotype-derived estimated breeding values (EBVs) [9–11] and/or marker-assisted or genomic selection [12, 13], where specific markers associated with the disease or genome-wide markers, respectively, are incorporated into breeding values. Previous studies regarding CHD, based on sample sizes of 150-800 animals and using microsatellites [14, 15] or SNPs [16–19], have shown inconsistent results for QTL position depending on the breeds, density of markers, allele frequencies and statistical methods used. These studies, usually focused on Norberg Angle or an equivalent measure to HS, have shown no evidence of a major locus controlling CHD, suggesting a complex disease driven by a number of QTL with small or moderate effect [17]. Although a few positional candidate genes have been proposed [19], only one study has demonstrated a putative association between mutations in a specific gene (fibrillin 2 gene) and the disease [20].

The purpose of this study was to perform genome- and chromosome-wide scans to identify QTLs for hip score and the three components associated with laxity in Labrador Retrievers, with the aims of improving selection schemes against the disease and furthering understanding of the biological basis of CHD.

## Methods

### Animals and phenotypes

The genotyped sample comprised 1500 hip-scored Labrador Retrievers born between 2002 and 2008, which provides a representative sample of animals used for breeding. Dogs were evaluated for hip dysplasia based on radiographs according to the UK scoring method, recording the nine hip score components for each separate hip [5]. These components are the following: Norberg Angle (NA), Subluxation (SUB), Cranial Acetabular Edge (CrAE), Dorsal Acetabular Edge (DAE), Cranial Effective Acetabular Rim (CrEAR), Acetabular Fossa (AF), Caudal Acetabular Edge (CAE), Femoral Head and Neck Exostosis (FHNE) and Femoral Head Recontouring (FHR). Owners of animals with hip scores and aged between 1 to 6 years old were contacted and requested to provide buccal DNA swabs and to fill in a questionnaire with details of body measurements (length, weight, girth and body conformation), exercise levels, lifestyle and nutrition, activity and concurrent health problems. Traits incorporated in statistical models are described below.

### Animal ethics

Radiographs were taken by veterinarians for submission to the British Veterinary Association/Kennel Club hip and elbow scoring schemes, a health screening protocol required before breeding from Kennel Club registered Labrador Retrievers. Hip and elbow score results are available to the public from the Kennel Club (https://www.thekennelclub.org.uk/services/public/mateselect/test/Default.aspx.).

Owners collected saliva samples themselves by using non-invasive buccal swabs after being provided with detailed instructions and an explanatory video. The sample was collected at home. This sampling strategy was chosen instead of involving a journey to a vet practice and collection by a veterinarian as this was deemed less stressful for the dog and of negligible risk. Advice obtained from personnel responsible for the ethical review process in The Roslin Institute (University of Edinburgh) was that no ethical approval was needed under the Animal Scientific Procedures Act (1986) because the technique was quick, non-invasive and painless and therefore was not a regulated procedure. The internal review process at the Institute also approved the research plan.

### DNA extraction and SNP genotyping

Extraction of DNA from buccal swabs was performed according to a standard protocol [21]. DNA was re-suspended in water and quantified using a Nanodrop and stored at 4°C until use. Animals were genotyped using the Illumina Canine High Density Beadchip containing 173,662 SNPs [22].

### Quality control procedures

**Figure 1:**
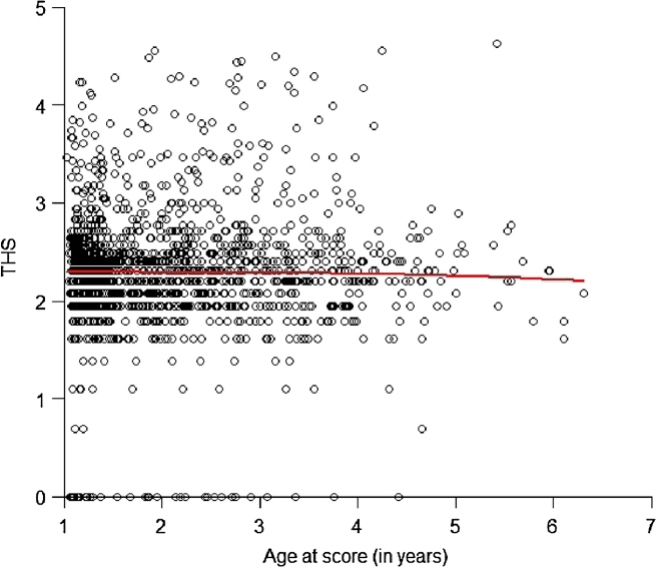
**Effect of the age at scoring on transformed hip score (THS).** The red trend line corresponds to a quadratic Loess regression.

Quality control was performed to assure both sample quality and marker quality [23]. A sample call rate threshold of 90% was applied, removing 275 samples with a low call rate. A further 27 animals were removed due to potential genotyping errors, detected as inconsistencies between the genomic and pedigree relatedness of individuals or between recorded sex and sex determined from the genotyping. The analysis of the age at scoring (Figure 1) showed a detrimental age effect on the hip score in animals older than 5 years. In order to remove this age-related bias, animals older than 5 years when scored (total of 19) were removed from the analysis, thus leading to a final sample size of 1179 animals.

Filtering criteria were applied to markers using Genome Studio software. A total of 59,260 markers were discarded due to low call rate (<98%), low reproducibility (GTS < 0.6), low or confounded signal (ABR mean < 0.3) and low minor allele frequency (MAF < 0.01). Further quality control on the markers was applied using PLINK [24], removing SNPs on the sex chromosomes and those showing deviation from Hardy-Weinberg equilibrium (threshold of 4.48E-7 applying a Bonferroni correction), finally resulting in 106,282 SNPs for further analysis, with their positions assigned according to the CanFam 2.0 assembly.

### Population structure

Preliminary analyses of population attributes (working status, coat colour) did not show associations with hip score, therefore, the genomic relationship matrix was fitted (as explained below) and was assumed to account for any remaining population structure.

### Genome-wide association analysis (GWAS)

Association analyses were performed for total hip score (HS), transformed total hip score (THS, logarithmic transformation, ln(1 + HS), following Lewis *et al*. [3]) and for the main three components on both hip sides (left, right and total): Norberg angle (NA), Subluxation (SUB) and Cranial Acetabular Edge (CrAE), using a linear mixed model as follows:


where ***y*** is the vector of phenotypes, ***W*** is a matrix of covariates with the **α** vector of associated fixed effects (including the intercept), ***x*** is a vector of marker genotypes (coded as 0/1/2) with *β* representing the regression of the phenotype on the marker genotypes, ***u*** is a vector of random polygenic effects and ***ϵ*** is a vector of residual errors. The fixed factors considered were the following: gender (1 degree of freedom), age at scoring in years and time spent exercising per day, which was scored from 1 (up to 1 hour) to 4 (more than 4 hours). The latter two factors were fitted as linear and quadratic regressions on deviations from their means. Analyses were conducted using GEMMA [25], which accounts for population stratification by including the genomic relatedness matrix (GRM, **G**) [26], and assuming a model where the vectors of random effects, ***u***, and errors, ***ϵ***, follow multivariate normal (MVN) distributions given by ***u*** *~* MVN(0,*V*_*G*_**G**) and **ϵ** ~ MVN(0,*V*_*E*_**I**), where *V*_*G*_ and *V*_*E*_ are the genetic variance associated with **G** and environmental variance, respectively.

GEMMA provides regression coefficient for each marker and their statistical significance was assessed using a Wald test. In determining a genome-wide significance threshold of P < 0.05, a conservative Bonferroni correction was made for multiple testing resulting from the large number of markers but not for multiple traits, resulting in a final threshold of P < 4.705E-7. The group of traits considered all share high pairwise genetic correlations, in excess of 0.85 [4, 6], and therefore the appropriate correction for multiple traits is unclear.

Differences in allele frequencies that are artefacts of cryptic population stratification or genotyping errors may inflate test statistics (mean and median χ^2^ values) above their expectations under the null hypothesis. This inflation was detected and corrected by the use of the inflation factor λ, defined as the ratio of the median of the empirically observed distribution of the test statistic to the expected median (thus quantifying the extent of the inflation and the excess false positive rate), following the method suggested by Amin *et al*. [27], assuming that the inflation is roughly constant across the genome.

### Genomic, chromosomal and regional variances

An improved estimate of *V*_*G*_, the total genetic variance, was obtained by removing the regression on marker genotype from the GWAS model. Therefore, the following mixed linear was fitted:


where the meaning and distributional assumptions for each of these terms were as described above for the GWAS model. To compare the estimates of genetic variance obtained from genomic and pedigree analyses, the model was re-fitted with ***u*** ~ MVN(0,*V*_*A*_**A**), where **A** is the numerator relationship matrix derived from the 5- generation pedigree of the phenotyped dogs, and *V*_*A*_ is the associated additive genetic variance.

Using similar models the genetic variance was also partitioned among chromosomes using two different approaches: (1) a joint decomposition of the 38 canine chromosomes involving the simultaneous fitting of 38 chromosome-specific GRMs, or (2) chromosome-specific analyses in which the GRM for each chromosome was fitted together with a complementary GRM constructed from the all other SNPs to account for the remaining polygenic effect. In the first approach, the model fitted was of the form:


where ***y*** is the phenotype of each individual and, as in previous models, ***W*** and **α** refer to the fixed effects described above. In this approach (joint chromosomal decomposition), each ***u***_(i)_ (i = 1, …, 38) represents a vector of genetic values for chromosome *i*, such that ***u***_(i)_ ~ MVN(0,*V*_*C(i)*_**G**_(i)_) where **G**_(i)_ is the GRM calculated from only those SNPs on chromosome *i*, and V_C(i)_ is the associated variance. The estimate of total genetic variance is  In the second approach, the total genetic variance was decomposed into a model with only two components:


where ***u***_(i)_ is the effect of chromosome *i*, as described above, and distributed as MVN(0,*V*_*C(i)*_**G**_(i)_) and ***u***_(‒i)_ is a polygenic effect for the remaining genome and distributed as MVN(0,*V*_*C(-i)*_**G**_(-i)_) where **G**_(-i)_ is a GRM constructed from all SNPs other than those on chromosome *i*. Therefore in this second model, the model was fitted 38 times, once for each of the 38 chromosomes, with analysis of chromosome *i* providing an estimate for *V*_*C(i)*_.

Regional *V*_*G*_ [28] for windows of 20 SNPs centred upon the GWAS-significant SNPs were evaluated considering the region plus a complementary polygenic effect. Regional *V*_*G*_ across the entire genome was also evaluated. In the latter analysis, the genome was divided into distinct regions, each containing 20 adjacent SNPs. The analysis then proceeded similarly to the approach described above for chromosomal variances with two components. Under the first approach, the SNP windows were always centred at the GWAS-significant SNPs, whereas under the second one, the significant SNP was not necessarily at the centre of a window.

The regional variances for significant SNPs and genomic and chromosomal variances were estimated for each trait using ACTA [29], but the full regional exploration with 20-SNP windows was carried out using REACTA [30] for computational ease.

An additional analysis of the haplotype structure and linkage disequilibrium (LD) in the genome-wide significant regions was performed using Haploview 4.2 [31]. Genome-wide significant regions were analysed for their haplotype structure and LD, and investigated for functional candidate genes showing strong LD with the significant SNPs.

## Results

**Figure 2:**
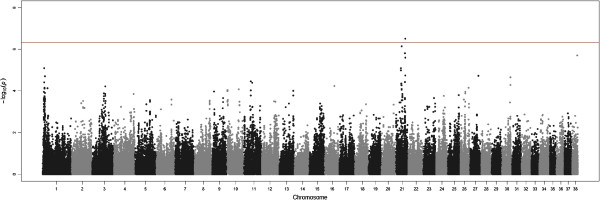
**GWAS analysis for the Norberg Angle right.** The genome-wide threshold (red line) corresponds to the Bonferroni correction for a nominal P-value = 0.05.

**Figure 3:**
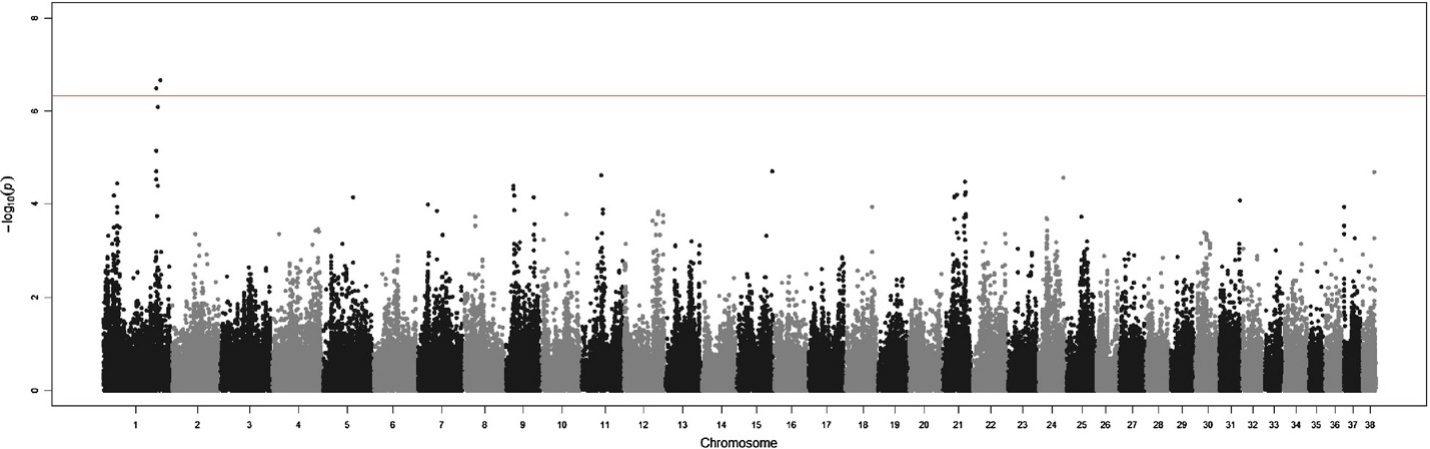
**GWAS analysis for the CrAE total.** The genome-wide threshold (red line) corresponds to the Bonferroni correction for a nominal P-value = 0.05.

Four SNPs with genome-wide significance (Table 1) and 73 SNPs with chromosome-wide significance (Additional file 1) were detected in the GWAS analyses and Manhattan plots for two of the traits are shown (Figures 2 and 3). The genomic estimates of the residual (*V*_*E*_) and genetic (*V*_*G*_) variances for each trait are shown in Table 2, together with the pedigree-based estimates. Quantile-Quantile (Q-Q) plots of GWAS analyses for all traits after correcting by the inflation factor are shown in the supplementary material (Additional file 2). The supplementary material also contains the estimates of the genetic variances (*V*_*C*_) at the chromosome level calculated using the two different approaches described above: joint chromosomal decomposition of the 38 chromosomes (Additional file 3) and separate analyses of each chromosome with all other chromosomes included as part of the polygenic effect (Additional file 4). Additional file 5 shows both sets of results in a graphical form. Results for the chromosomal analyses (Additional files 3 and 4) are presented as percentages of the total genetic variance explained by the genomic estimates given in Table 2 and thus add up to 1. The regional heritability analysis determined percentages of genetic variance explained for each trait based on windows of 20 SNPs across the genome (Additional file 6) or only for the regions centered at the GWAS-significant SNPs (Additional file 7).

**Table 1:** **Summary of genome-wide significant SNPs using a linear mixed model for canine hip dysplasia in UK Labrador retriever**

CHR	SNP	Position	Trait	MA	MAF	Beta coef.	P-value	%V _G_
1	BICF2P219706	100106009	CrAE left	G	0.019	0.52 ± 0.10	3.35E-07	22.2 ± 17.2
1	BICF2P219706	100106009	CrAE total	G	0.019	0.94 ± 0.18	3.20E-07	13.5 ± 9.90
1	BICF2S2443186	100138261	CrAE left	A	0.019	0.52 ± 0.10	3.34E-07	22.6 ± 17.7
1	BICF2S2443186	100138261	CrAE total	A	0.019	0.94 ± 0.18	3.21E-07	13.8 ± 10.4
1	BICF2P1285984	107719908	CrAE left	G	0.018	0.53 ± 0.10	1.97E-07	33.2 ± 19.5
1	BICF2P1285984	107719908	CrAE total	G	0.018	0.97 ± 0.19	2.20E-07	20.6 ± 11.8
21	BICF2P429643	43337454	NA right	G	0.222	0.40 ± 0.08	3.08E-07	13.1 ± 9.1

The table shows chromosome, significant SNPs, position (in base pairs according to CanFam 2.0), associated trait (total hip score (HS), transformed total hip score (THS), Norberg Angle (NA), Subluxation (SUB) and Cranial Acetabular Edge (CrAE)), minor allele (MA) and its frequency (MAF), Beta coefficient (minor allele substitution effect), P-value for the Beta coefficient from the GWAS analysis and percentage of genetic variance explained by a region of 21 SNPs centred at the significant SNP.

**Table 2:** **Estimates of variance components**

Trait	Pedigree			Genomic		
	h ^2^	V _G_	V _E_	h ^2^	V _G_	V _E_
**HS**	0.59 ± 0.13	73.13 ± 17.33	51.12 ± 15.08	0.23 ± 0.06	27.42 ± 7.41	93.57 ± 7.22
**THS**	0.27 ± 0.11	0.11 ± 0.04	0.29 ± 0.04	0.25 ± 0.06	0.10 ± 0.02	0.29 ± 0.02
**NA right**	0.29 ± 0.11	0.56 ± 0.22	1.41 ± 0.21	0.15 ± 0.05	0.30 ± 0.11	1.97 ± 0.08
**NA left**	0.52 ± 0.12	1.10 ± 0.27	1.01 ± 0.24	0.36 ± 0.06	0.66 ± 0.14	1.43 ± 0.12
**NA total**	0.44 ± 0.12	2.88 ± 0.81	3.65 ± 0.73	0.27 ± 0.06	1.74 ± 0.40	4.73 ± 0.38
**SUB right**	0.28 ± 0.10	0.29 ± 0.10	0.77 ± 0.10	0.21 ± 0.06	0.22 ± 0.06	0.84 ± 0.06
**SUB left**	0.23 ± 0.10	0.33 ± 0.12	0.78 ± 0.11	0.18 ± 0.06	0.20 ± 0.06	0.90 ± 0.07
**SUB total**	0.36 ± 0.10	1.09 ± 0.33	1.95 ± 0.31	0.28 ± 0.06	0.85 ± 0.19	2.19 ± 0.13
**CrAE right**	0.19 ± 0.10	0.08 ± 0.04	0.32 ± 0.04	0.20 ± 0.06	0.08 ± 0.02	0.32 ± 0.02
**CrAE left**	0.06 ± 0.08	0.03 ± 0.04	0.41 ± 0.04	0.11 ± 0.05	0.05 ± 0.02	0.40 ± 0.03
**CrAE total**	0.15 ± 0.10	0.21 ± 0.14	1.23 ± 0.14	0.18 ± 0.06	0.26 ± 0.08	1.18 ± 0.09

The table shows the heritabilities (*h* ^*2*^) and genomic (*V*_*G*_) and residual variances (*V*_*E*_) estimated under the pedigree and genomic approaches for each trait (total hip score (HS), transformed total hip score (THS), Norberg angle (NA), Subluxation (SUB) and Cranial Acetabular Edge (CrAE)).

### Hip score (HS) and Transformed hip score (THS)

No SNPs with genome-wide significance were detected for these traits (Table 1), although the application of chromosome-wide thresholds (Additional file 1) identified several significant SNPs for both HS (on Chr 1, 2, 11, 15, 21 and 23) and THS (on Chr 11, 24 and 38).

Chr 9 and 11 clearly explained the highest proportions of genetic variance obtained from the joint chromosomal decomposition for HS (22.6% and 19.7% of the total *V*_*C*_). In contrast, for THS, *V*_*C*_ was more evenly distributed across the genome, with Chr 1, 9, 21 and 24 explaining the highest proportions of *V*_*C*_, 6.6%, 15.3%, 11% and 8.3%, respectively (Additional files 3, 4 and 5).

The estimates of the residual variance (*V*_*E*_) from genomic analyses were greater (and heritability lower) than their pedigree estimates (Table 2) for HS, indicating that the GRM did not explain as much of the observed phenotypic variance and thus appeared not to capture the full additive genetic variance. In contrast, for THS, the estimates of *V*_*E*_ were very similar. The regional heritability analyses (Additional file 6) showed several peaks across the genome, and although no significant regions were detected for HS, a significant region was detected for THS on Chr 21 (41.4 Mb). This region was not concordant with the chromosome-wide significant SNP detected by GWAS for this trait (27.9 Mb) but close to significant SNPs detected for other traits (NA and CrAE) on this chromosome.

### Norberg Angle (NA)

A SNP with genome-wide significance (Table 1 and Figure 2) was detected for the right hip on Chr 21 and several SNPs with chromosome-wide significance (Additional file 1) were detected for the right hip (Chr 1, 10, 12, 13, 15, 21, 26, 27, 30 and 38), the left hip (Chr 4, 14, 22, 23 and 25) and the total NA (Chr 12, 21, 23, 27 and 38).

For the right-hip NA, Chr 11 and 21 clearly explained the highest proportions of genetic variance (21.7% and 16.9% of *V*_*C*_) whereas for the left-hip NA, the total *V*_*G*_ was more evenly distributed across the genome, with several chromosomes explaining similar percentages. Results for total NA were a mixture of those for left and right hips, with Chr 3, 4, 9, 11 and 21 explaining the highest proportions of *V*_*C*_, 6.7%, 6.9%, 6.8%, 7.5% and 6.7%, respectively (Additional files 3, 4 and 5).

The estimates of residual variances (*V*_*E*_) from genomic analyses for all three traits associated with NA were greater (with lower heritability) than the pedigree estimates (Table 2), again indicative that the GRM did not capture the full genetic variance. Regional heritability analyses showed several peaks across the genome, none of which were significant (Additional file 6). The SNP on Chr 21 detected by GWAS explained 13.1% of the genetic variance (*V*_*G*_) but the standard error was very high (Table 1).

### Subluxation (SUB)

No SNPs with genome-wide significance were detected, although several SNPs with chromosome-wide significance (Additional file 1) were detected for the right hip (Chr 6, 22, 24, 31 and 38), the left hip (Chr 6, 20 and 36) and total SUB (Chr 6, 24, 27 and 35).

For the right-hip SUB, Chr 1, 7, 9, 21 and 24 explained the highest proportions of total genetic variance, 9.7%, 8.2%, 7.5%, 8.3% and 8.7% of the total *V*_*G*_, respectively, whereas for the left-hip SUB, the highest proportions were explained by Chr 9 and 24 (10% and 11%). In the case of SUB total, Chr 1, 9 and 24 explained the highest proportions, 7.2%, 7.2% and 7.3% of the total *V*_*G*_, respectively (Additional files 3, 4 and 5).

The estimates of residual variances (*V*_*E*_) from genomic analyses for all three traits were again slightly higher (lower heritability) than the pedigree estimates (Table 2), and regional heritability analyses showed several non-significant peaks and one significant peak on Chr 9 for left-hip SUB (16.5 Mb), which was not concordant with SNPs detected for other traits (Additional file 6).

### Cranial Acetabular Edge (CrAE)

SNPs with genome-wide significance (Table 1 and Figure 3) were detected on Chr 1 for the right and the left hips, and several SNPs with chromosome-wide significance (Additional file 1) were also detected for the right hip (Chr 13 and 21), the left hip (Chr 12, 15 and 38) and the total CrAE (Chr 12 and 38).

Unlike HS and traits associated with NA and SUB, the genomic estimates of the residual variances (*V*_*E*_) for the three traits were very close to (and slightly lower than) pedigree estimates (Table 2). The regional heritability analyses did not reveal any significant peaks across the genome (Additional file 6). The SNPs detected by GWAS on Chr 1 showing genome-wide significance explained 13.5-33.2% of the genetic variance (*V*_*G*_) but again the standard errors were very high (Table 1).

### General observations across traits

Due to the Beavis effect, the estimates of effect sizes (Beta coefficients) and the proportion of genetic variance explained by each chromosome or region, considered separately, are expected to be overestimates [32]. Therefore the results obtained by the joint chromosomal decomposition (fitting all the chromosomes simultaneously in the same model) are expected to be more accurate than the chromosomal variances obtained for each chromosome separately when compared with the total (GRM-based) genetic variance. The similarity of our estimates of *V*_*G*_ and *V*_*C*_ calculated from the joint chromosomal decomposition (results not shown), support this prediction and therefore, we subsequently focus on estimates of individual chromosomal variance from the joint chromosomal decomposition. These estimates revealed a polygenic architecture for all traits, with several chromosomes contributing less than 6% of the total genetic variance and a few chromosomes contributing 15-23%.

All animals, including those with perfect hip scores (HS = 0), were included in the analyses. However, Lewis *et al*. [4] detected an excess of zero individual scores for HS, based on non-linearity of the regression of offspring phenotype onto mid-parent phenotype, suggesting a lower precision of evaluation in the lower tail of the trait distribution. To address this, parallel genome-wide GWAS analyses were performed for all traits removing all animals with a HS = 0. Results of these analyses (not shown) were similar to the results shown previously for the full sample of animals.

## Discussion

The present study used a high density canine chip to identify two genome-wide QTLs affecting CHD-related traits. Chr 1 and 21 revealed consistent GWAS peaks and significant SNPs across the analyses in relation to several traits. Other suggestive QTLs were detected as significant at the chromosome-wide level, and may also indicate genomic regions related to hip dysplasia. In order to strengthen the evidence for QTLs identified in our study, we have applied two additional analyses: chromosomal and regional analyses of the explained genetic variance. These methods are designed to estimate variances associated with multiple loci contributing localised variance that may be too small individually to detect by single association tests [28, 33] and to estimate the variance explained by a QTL rather than its allelic substitution effect.

Chr 1 explained ~23% and ~13% of the total *V*_*G*_ observed for CrAE left and CrAE total, respectively, and revealed six genome-wide significant SNPs for these traits, as well as chromosome-wide significant SNPs related to HS and NA right. These SNPs were located between two QTLs for CHD status in German Shepherds [16] and within 10 Mb of a QTL for NA right in Portuguese Water dogs [14]. Genes in the region between 99.00 Mb and 110.00 Mb on Chr 1, which encompasses all of the genome- and chromosome-wide significant SNPs, were investigated. Two genome-wide significant SNPs were found within the *SHC3* gene, which is not known to be associated with CHD-related traits, however, within 1 Mb of this gene, there were several functional candidate genes, associated with bone formation or mineralization (*SEMA4D, OMD, OGN*), cartilage formation (*PHF2*), and differentiation into joint versus cartilage cells (*BARX1*). However, none of the SNPs within or closest to these genes showed high LD (assessed by *r*^2^) with the SNPs identified by GWAS. The third genome-wide significant SNP was found in an inter-genic region, close to a zinc-finger gene (*ZNF677*), which again is not known to have any association with CHD-related traits, and several uncharacterised protein-coding genes. The large size of the Chr 1 region, and hence the large number of positional candidate genes, highlights a problem in the identification of causative variants from GWAS studies in species with high levels of LD such as dogs.

While this 11-Mb region on Chr 1 included the greatest number of genome-wide significant SNPs in our study, the frequency of the favourable SNP alleles (associated with low hip score) is already very high, suggesting that the average effect of the favourable allele is small [34] and consequently little genetic progress in CHD could be made by changes in frequencies at this QTL. This assumes that the allele frequency of the causal variant is similar to the marker, which may not be the case. However, the *r*^*2*^ (and power of detection) between a marker and a causal variant is sensitive to differences in allele frequency, particularly at low frequencies, suggesting that the allele frequencies of the marker and causative variant cannot be radically different.

Chr 21 explained ~17% of the total *V*_*G*_ observed for NA right, revealing one genome-wide significant SNP for this trait, several chromosome-wide significant SNPs related to NA, CrAE and HS and a significant peak in the regional analysis for THS. These SNPs correspond to two main regions: a genome-wide significant region situated at ~43 Mb and a chromosome-wide significant region situated at ~27 Mb. For the first case, previous studies have shown the existence of a QTL related to NA in this region for a Labrador-Greyhound cross-breed pedigree [17] and ~5 Mb from a QTL for CHD status in German Shepherds [16]. The genome-wide significant SNP from our study is found within the *OTOG* gene, which encodes an ear-specific glycoprotein that has no known association with CHD-related traits. However, as with the Chr 1 region, there are several functional candidate genes between 41.5 and 44.5 MB, the region that includes the genome- and chromosome-wide significant SNPs. These include genes associated with cartilage formation (*SOX6*), inflammation and osteoarthritis (*SAA*) and muscle cell differentiation and muscle regeneration (*MYOD1, SERGEF*). Again, most of the SNPs within or closest to these genes showed low LD with the SNPs identified by GWAS, however, one SNP within *SERGEF* was strongly correlated (*r*^2^ = 0.8) with the genome-significant SNP and two SNPs within *SOX6* were moderately correlated with it (*r*^2^ = 0.4). In comparison to the region on Chr 1, there is greater potential for genetic progress based on this region as the frequency of the favourable allele is much lower for this QTL.

Chromosome-wide QTLs were detected on several chromosomes and a polygenic pattern was also observed for the distribution of the genetic variance in both the regional and chromosomal studies, with most of the chromosomes explaining less than 6% of the genetic variance but some chromosomes explaining between 10% and 23%. These results confirm a polygenic nature of the disease that could arise if the hip score and its components were the result of multiple interacting processes. It might be the case that genes affecting one or two processes have a small effect on the main phenotype (resulting in many QTLs with small effect), while only genes affecting several processes appear as QTLs with larger effects. The complex aetiology of the disease supports its polygenic nature. Both biological and environmental factors potentially contribute to an unstable hip joint [35] and many different genes are likely to influence the biological factors (e.g. bone and cartilage formation, muscle and other soft tissue development, inflammatory responses), which when disrupted, could contribute to disease. It is worth noting that while identification of the genes associated with the major QTLs detected in our study would contribute to an improved understanding of the biology of CHD and may help in the development of therapies, our results suggest that these QTLs will not capture most of the genetic variance related to this disease.

Another aspect of the trait complexity found in our study and several others [14, 15, 17, 36] is that some QTL effects are stronger on one side of the body than the other, despite the fact that a high genetic correlation between right and left scores has been observed in both Labrador Retriever and German Shepherd breeds [4, 37]. These findings could result from asymmetrical dogs’ gaits (even with normal hips) [38] or from suboptimal positioning of dogs when scoring, but also could be a statistical artefact of identification of QTL with small effects.

In order to further explore the QTLs identified in our study, the performance of the SNP-based genomic approach to characterise the genetic architecture of the trait was also compared with the pedigree approach in terms of residual variance (*V*_*E*_). Residual variance was used to compare the models because in both cases, it comprises the variance component not explained by the model for the phenotypes in the data, whereas, in contrast, the genetic variances obtained using genomic and numerator (pedigree-based) relationship matrices refer to constructed base populations, which may differ. SNP-based methods are not expected to capture all the genetic variance [33] and our results broadly confirm this hypothesis, with our genomic estimates of *V*_*E*_ greater than those provided by a pedigree analysis for most of the traits, but not in all cases. It is expected that an increase in the number of markers will increase the proportion of explained genetic variance [39, 40].

Although some detected QTL regions (e.g. Chr 1 and 21) correspond with those found in previous QTL studies, others have not been seen before and equally, we did not detect effects in several previously identified regions. There was no overlap between the regions showing genome- or chromosome-wide significance and those associated with CHD severity in a recent case-control study of 78 Dutch Labrador Retrievers [41] (the closest SNPs detected in their study, on Chr 25, were > 5Mb from a chromosome-wide significant region in our study). This inconsistency of QTLs across different studies has been previously pointed out by Zhu *et al*. [17], and in addition to the possibility that some results are false positives, might be explained by the different selection pressures applied to each breed or population, the varying LD patterns found in different breeds [42, 43], the specific details of the different studies (number of animals, pedigree structure, marker type and density) and the stochasticity associated with detecting small (but real) effects across multiple regions. Some of these factors probably also account for the limited consistency between GWAS results for the highly correlated traits within this study.

## Conclusion

In summary, two genome-wide significant QTLs on Chr 1 and 21 and three chromosome-wide significant QTLs on Chr 11, 21 and 24 were detected in a Labrador Retriever population, associated with hip score or its components. However, none of the chromosomes explained more than 23% of the genetic variance of the traits. These findings, taken with the complex nature of the hip score as the sum of different components, possibly related to different metabolic processes, suggests a genetic architecture based on many genes with small or moderate effect and therefore, marker-assisted selection is not likely to be successful. Alternatively, a genomic selection approach against the disease should be considered [44].

## Electronic supplementary material

Additional file 1: **Summary of chromosome-wide significant SNPs using a linear mixed model for canine hip dysplasia in UK Labrador Retriever:** The table shows chromosome, significant SNPs, position (in base pairs according to CanFam 2.0), associated trait, minor allele and its frequency, Beta coefficient (minor allele substitution effect), P-value of the GWAS analysis and percentage of genetic variance explained by a region of 21 SNPs centred in the significant SNP. (XLSX 19 KB)

Additional file 2: **Quantile-Quantile plots for GWAS analyses.** Figures show the Q-Q plots after correcting by the inflation factor. (PDF 301 KB)

Additional file 3: **Percentage of genetic variance explained per chromosome: The joint chromosomal decomposition model fitted the 38 chromosomes simultaneously.** (Note: equivalent to the red bars in Additional file 5). Percentages are related to the total genetic variance explained by the genomic method (given in Table 2). The proportion of the genome accounted for by each chromosome (PG) is also given. (XLSX 11 KB)

Additional file 4: **Percentage of genetic variance explained per chromosome: The model considers a separate analysis of each chromosome including a complementary GRM accounting for the polygenic effect.** (Note: equivalent to the blue bars in Additional file 5). Percentages are related to the total genetic variance explained by the genomic method (given in Table 2). The proportion of the genome accounted for by each chromosome (PG) is also given. (XLSX 14 KB)

Additional file 5: **Percentage of genetic variance explained per chromosome.** Blue bars correspond to estimates analysing each chromosome separately (Additional file 4), and red bars correspond to estimates obtained through the joint chromosomal decomposition (Additional file 3). Error estimates for the first method (dashed lines) were obtained based on a Taylor series approximation. Lack of convergence of the REML analyses for the second method meant that the variance/covariance matrix, and thus the errors, could not be estimated. (PDF 897 KB)

Additional file 6: **Regional heritability analysis.** P-values for the significance of the genetic variance explained by each window of 20 SNPs for all traits, considering a Bonferroni-corrected threshold (red line). (PDF 1 MB)

Additional file 7: **Percentage of genetic variance explained for all significant SNPs.** Contains the percentage of genetic variance explained by all significant SNPs (% V_G_ SNPs) or by the regions of 20 SNPs centered at the significant SNPs (% V_G_ Regions). (XLSX 11 KB)
