# Supplementary figures and images for: Quantitative trait loci mapping for canine hip dysplasia and its related traits in UK Labrador Retrievers
Source: BMC Genomics. 2014 Oct 1;15(1):833. doi: 10.1186/1471-2164-15-833 (PMC4190382; doi:10.1186/1471-2164-15-833)

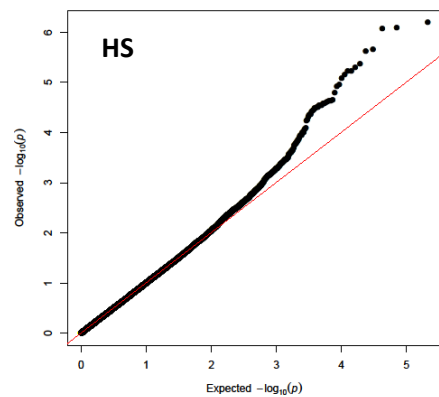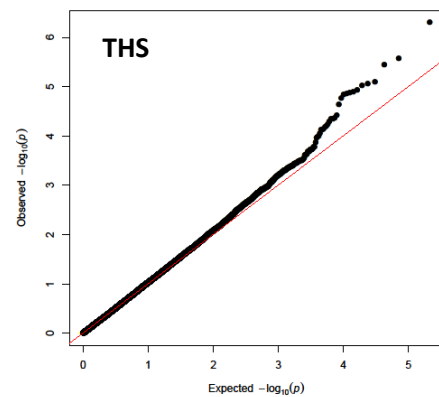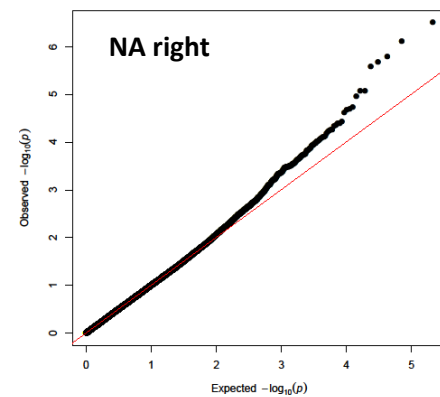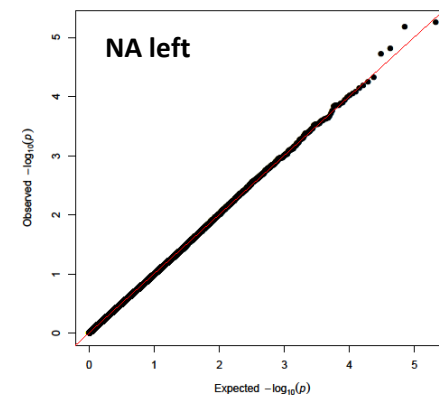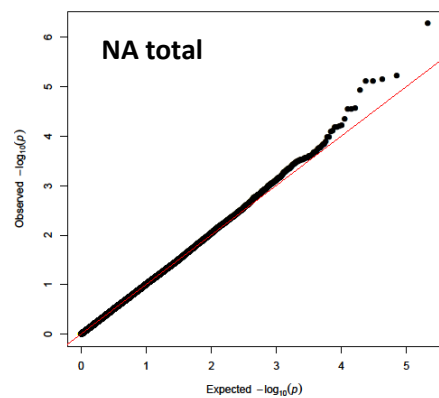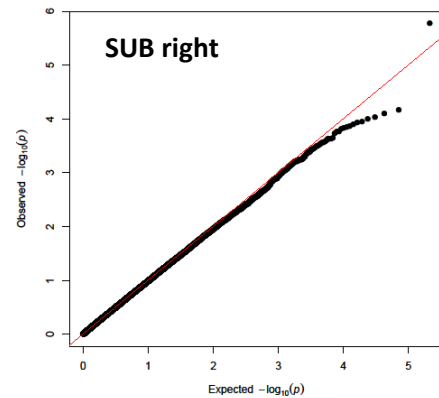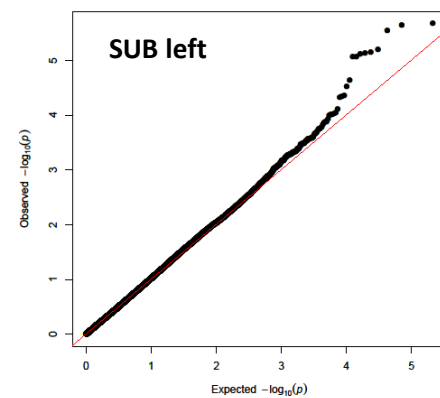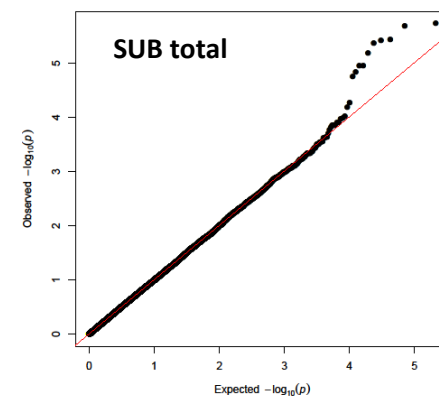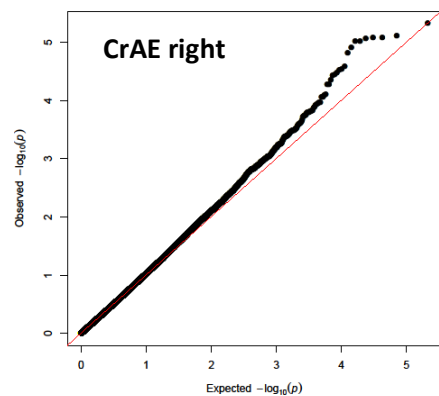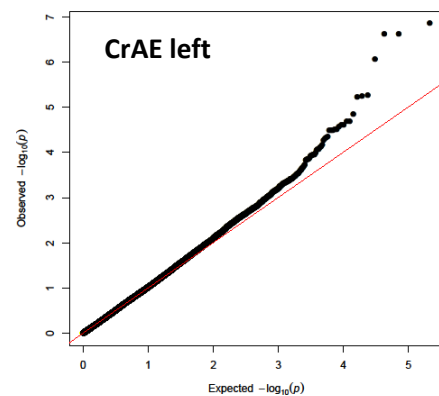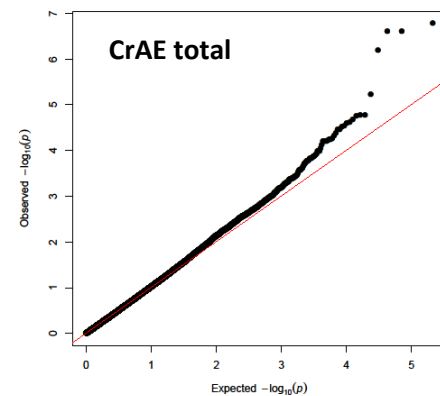

Supplement: Supplementary file 2 — Additional file 2: Quantile-Quantile plots for GWAS analyses. Figures show the Q-Q plots after correcting by the inflation factor. (PDF 301 KB) [file 12864_2014_6512_MOESM2_ESM.pdf]

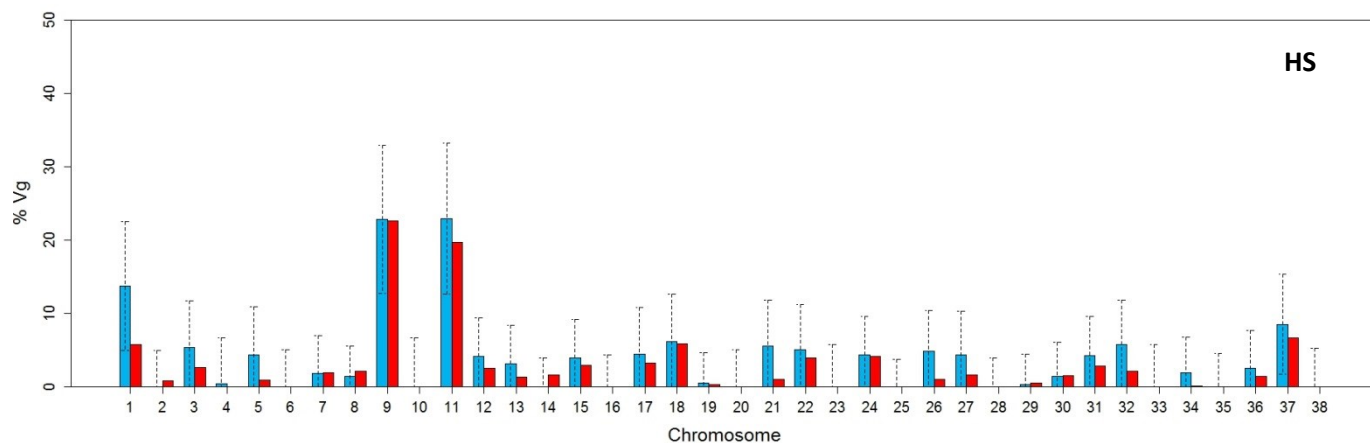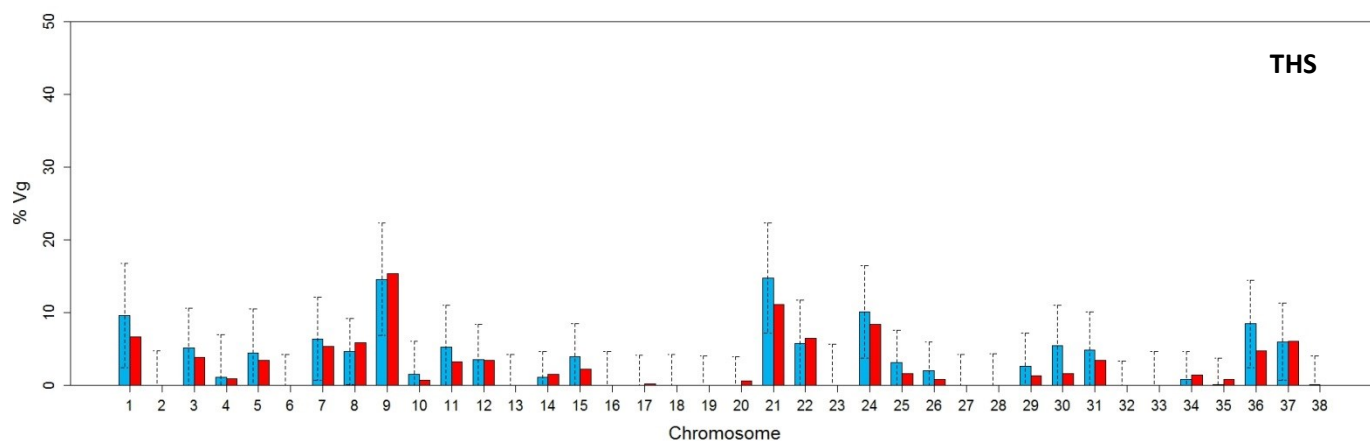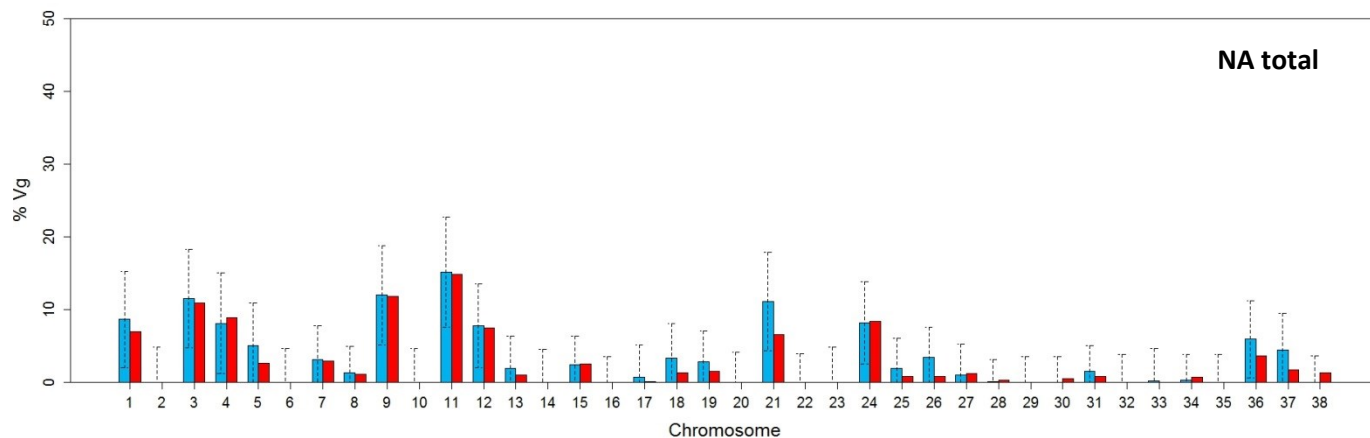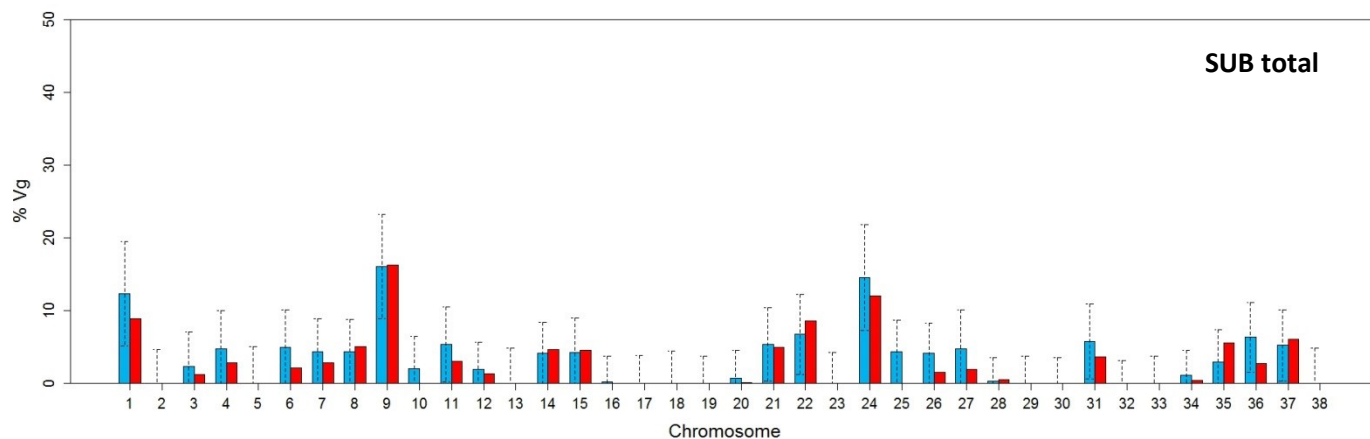

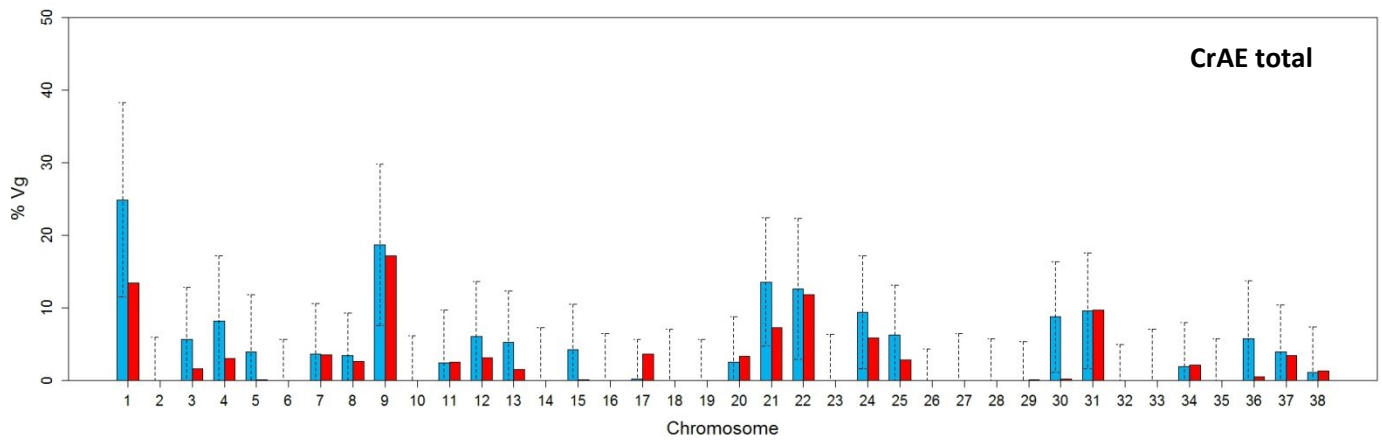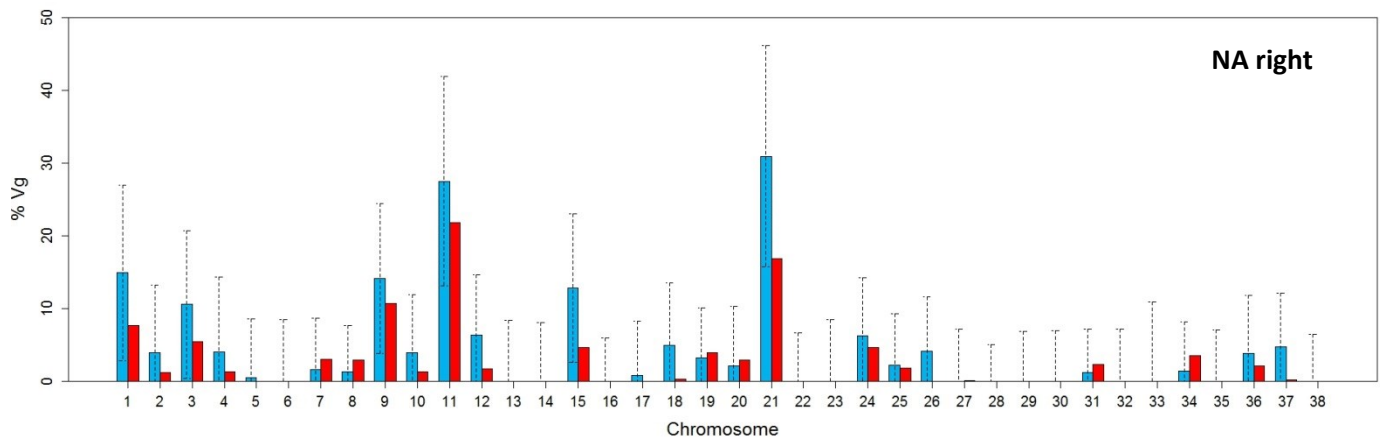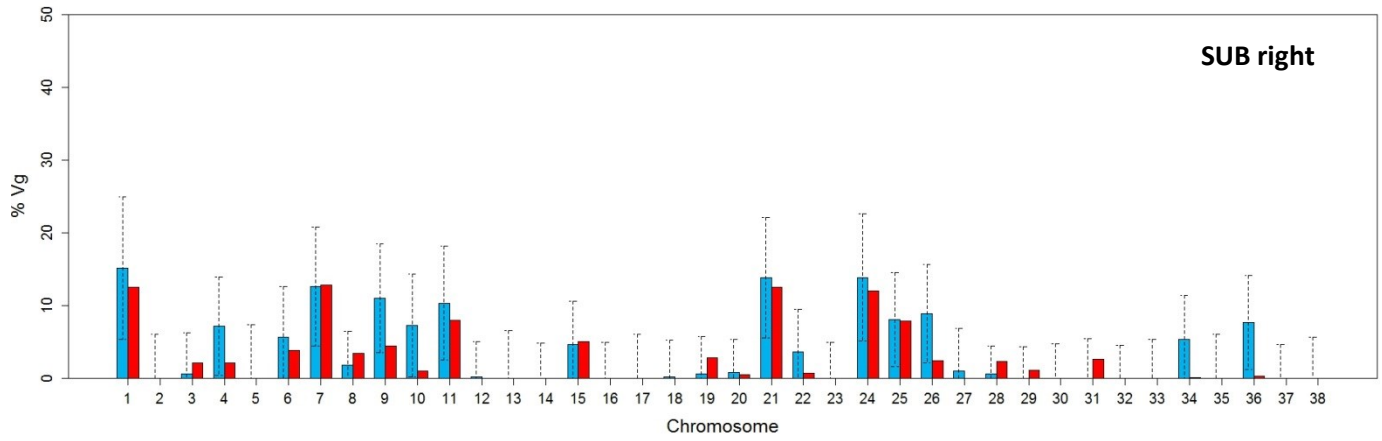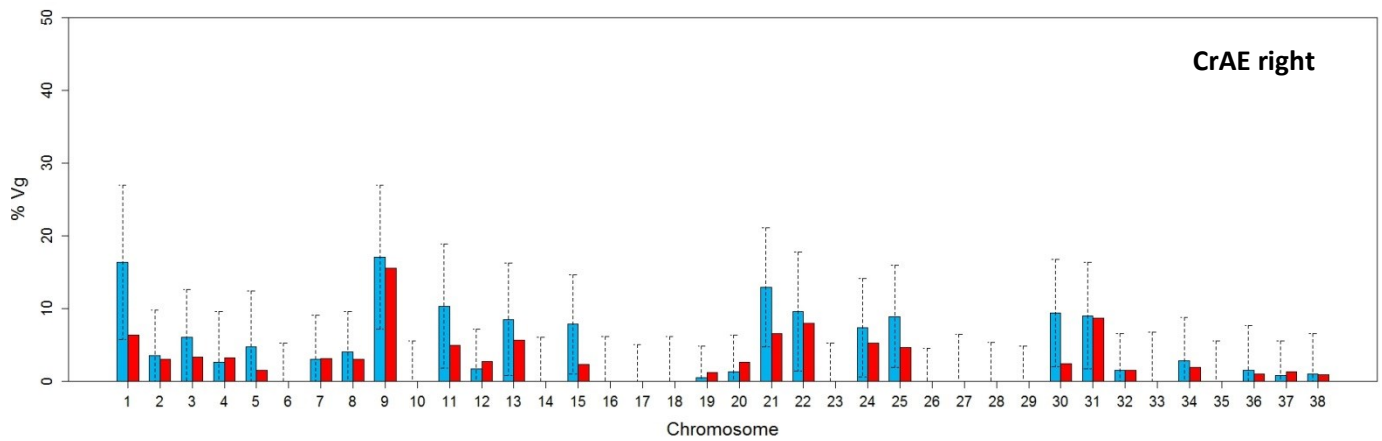

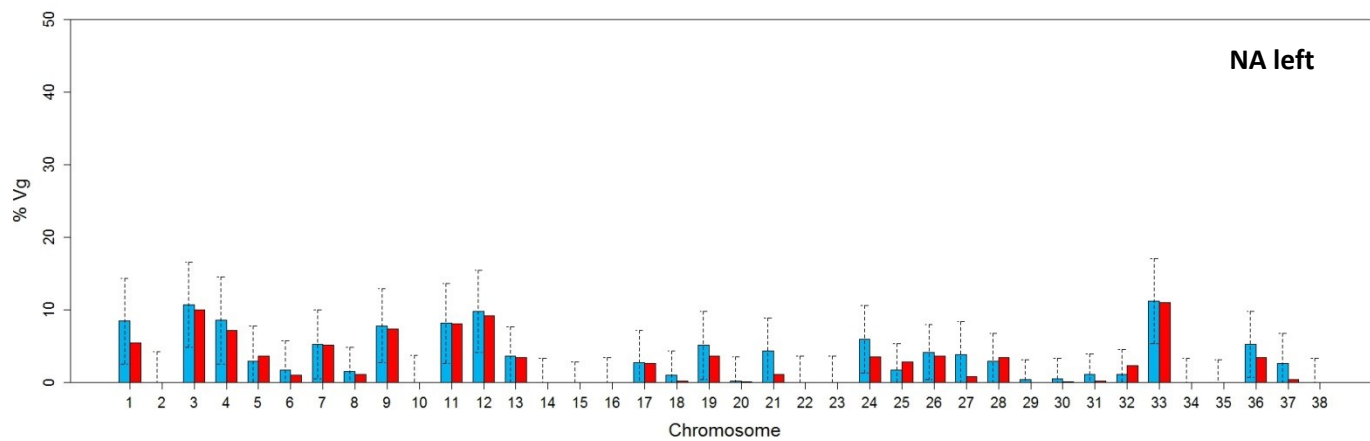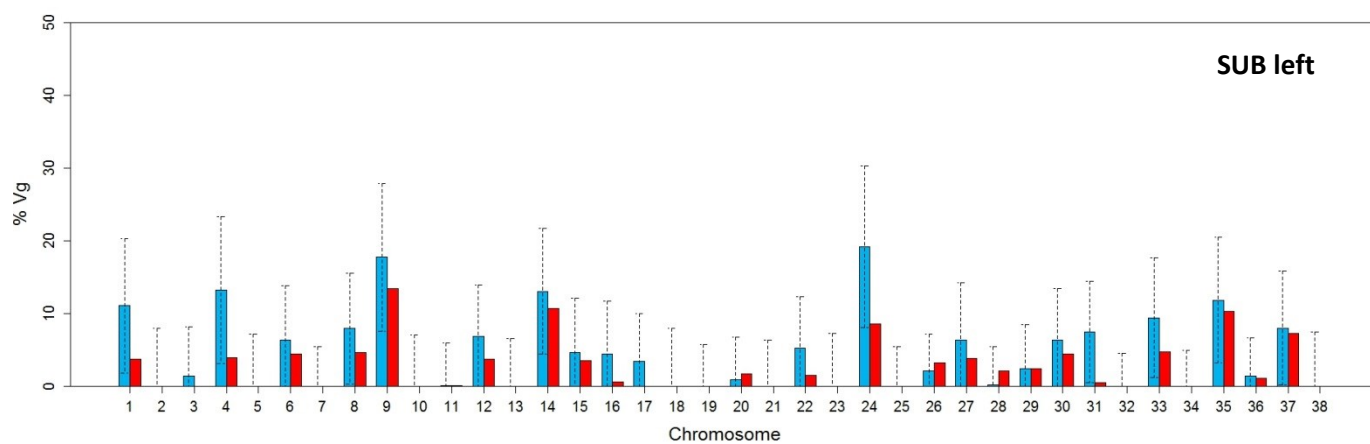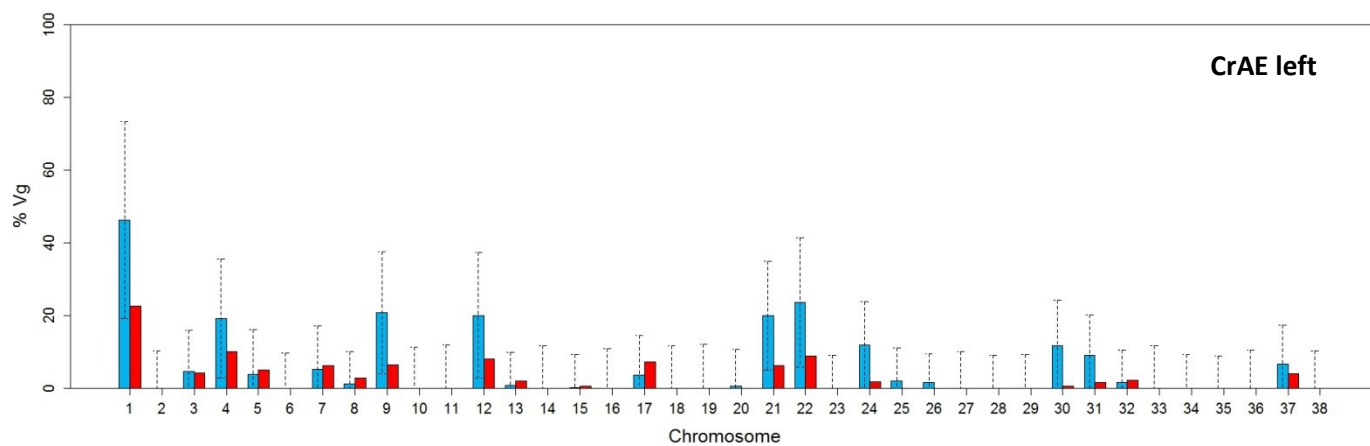

Supplement: Supplementary file 5 — Additional file 5: Percentage of genetic variance explained per chromosome. Blue bars correspond to estimates analysing each chromosome separately (Additional file 4), and red bars correspond to estimates obtained through the joint chromosomal decomposition (Additional file 3). Error estimates for the first method (dashed lines) were obtained based on a Taylor series approximation. Lack of convergence of the REML analyses for the second method meant that the variance/covariance matrix, and thus the errors, could not be estimated. (PDF 897 KB) [file 12864_2014_6512_MOESM5_ESM.pdf]

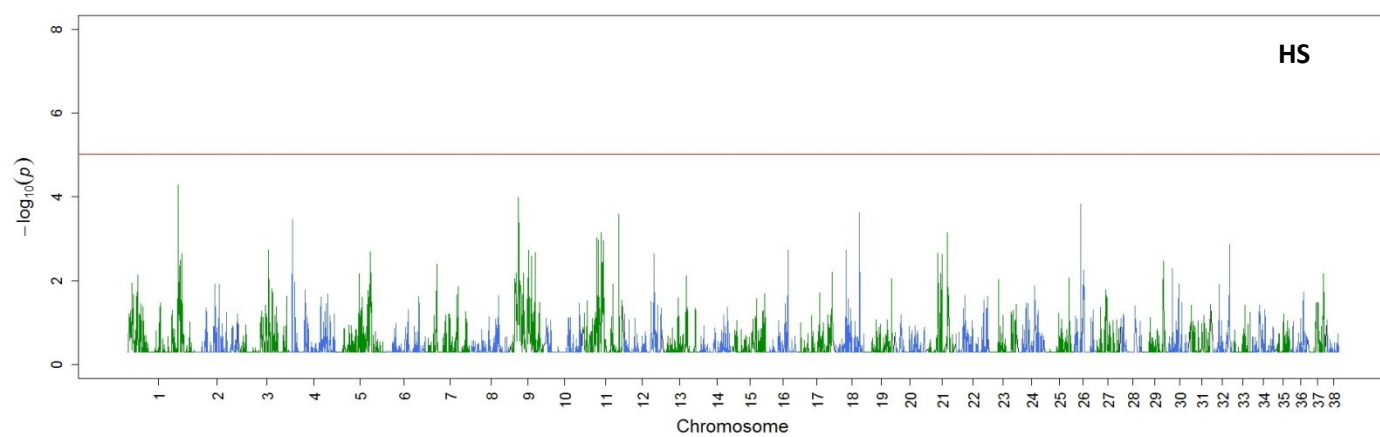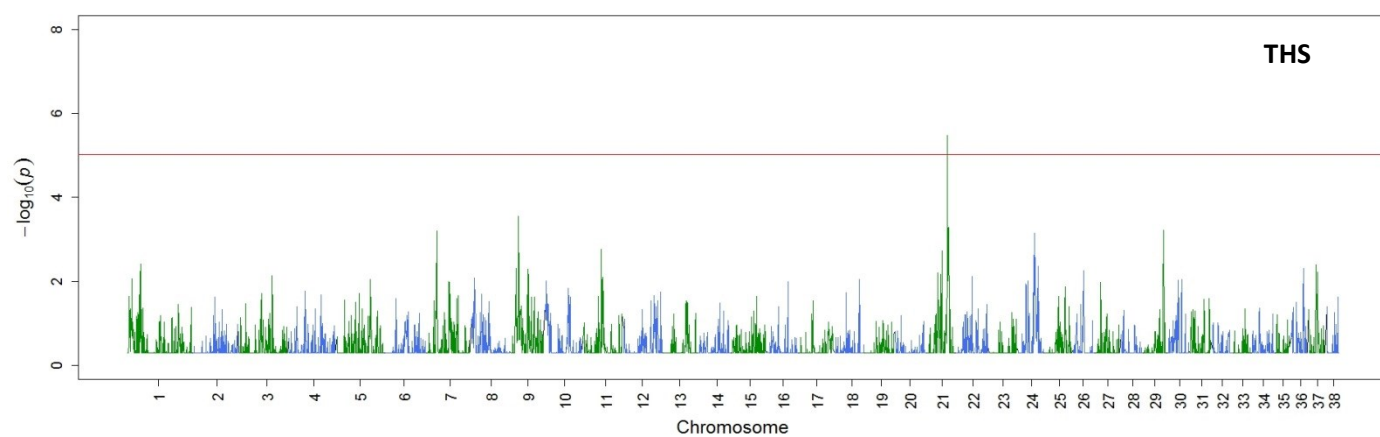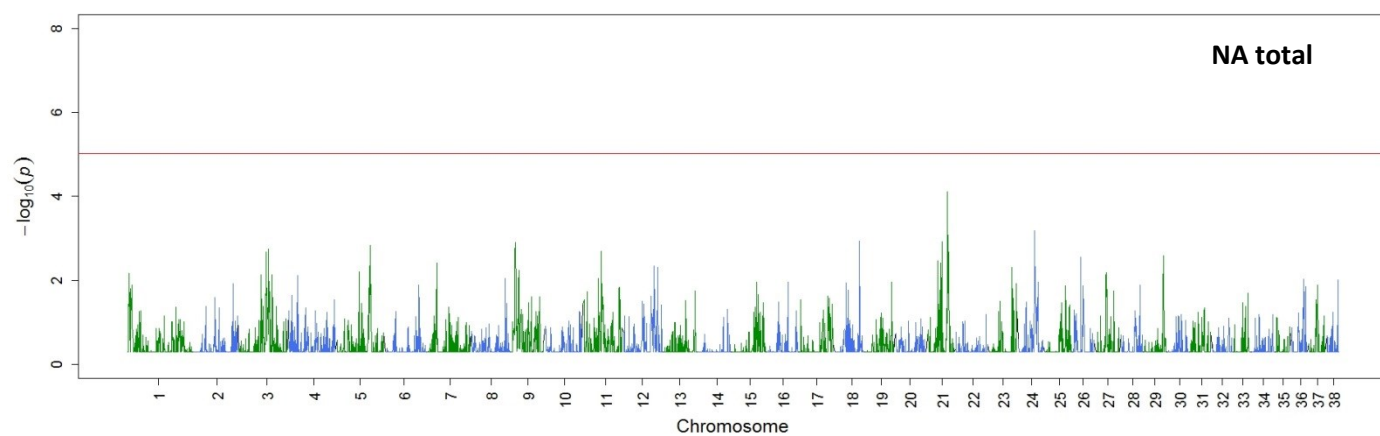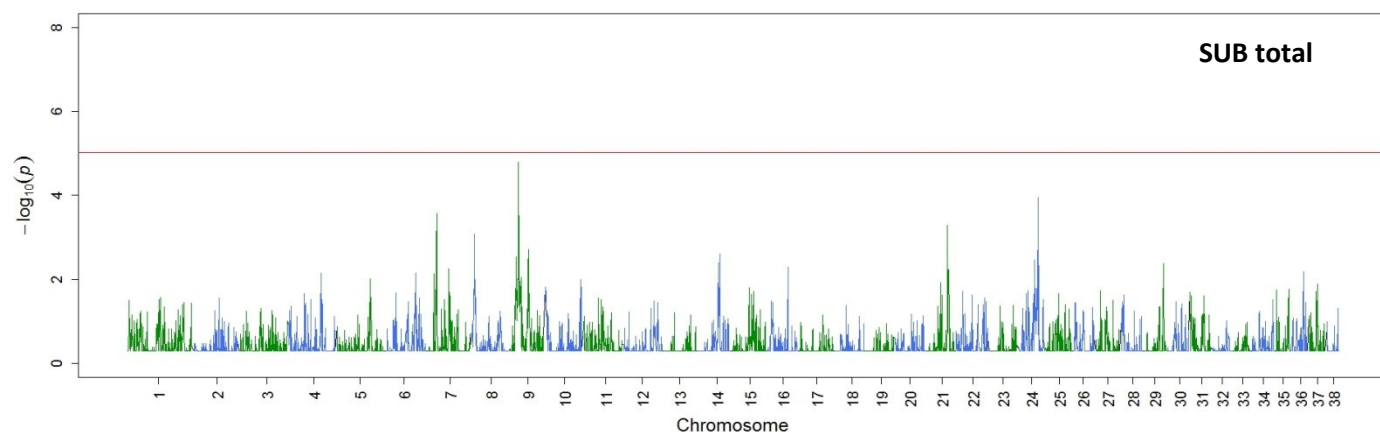

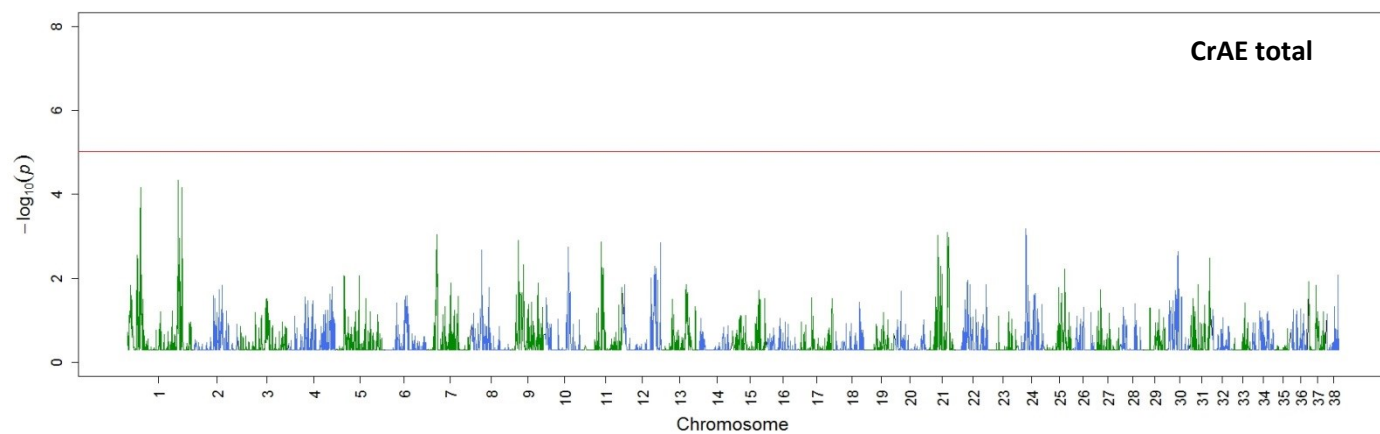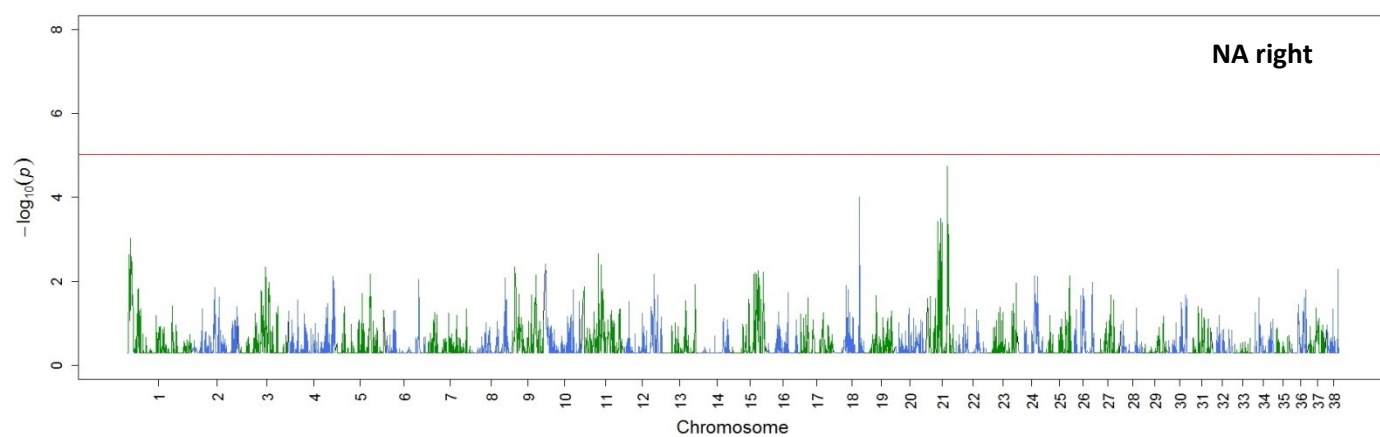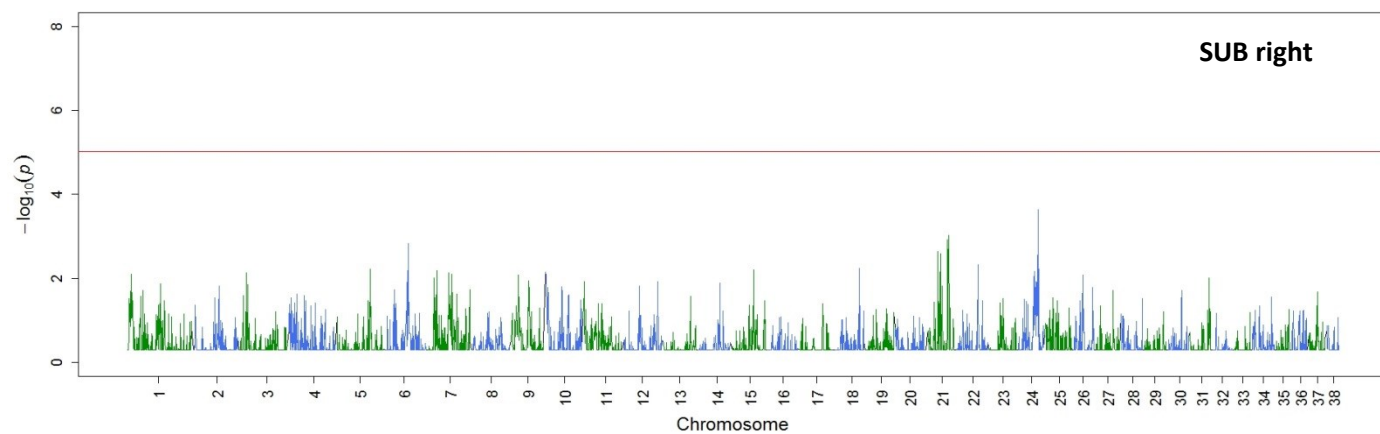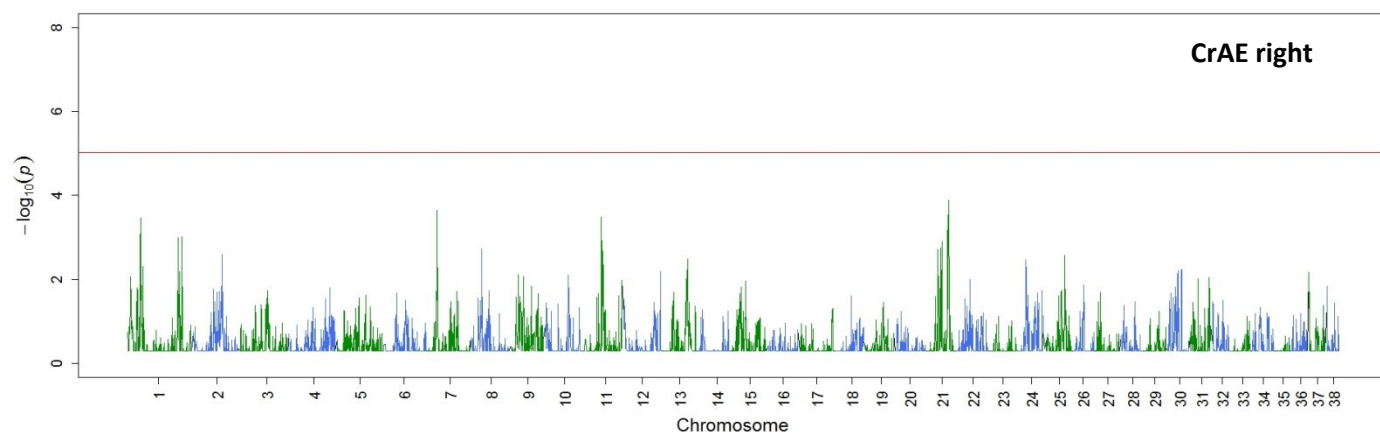

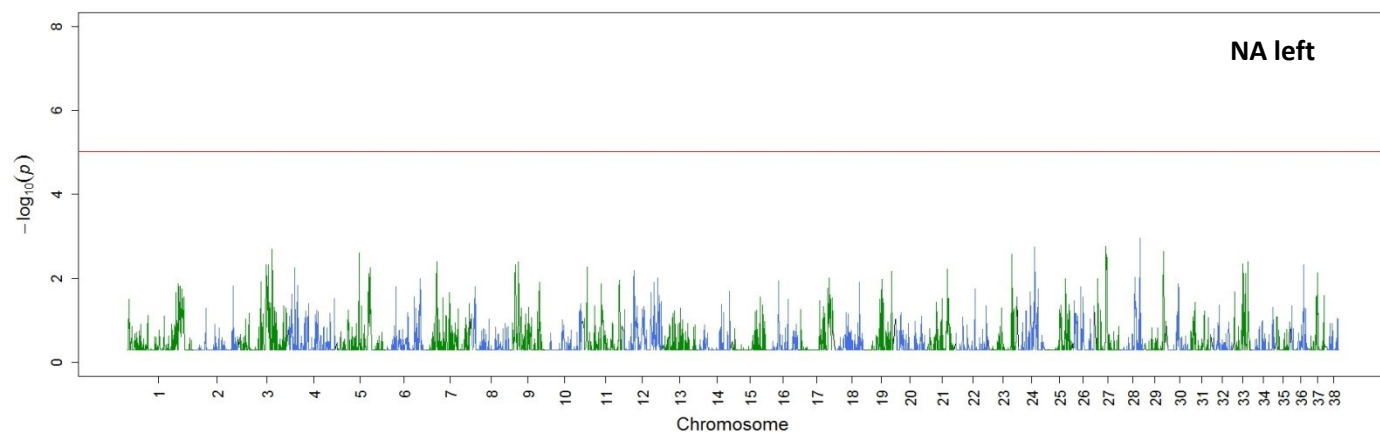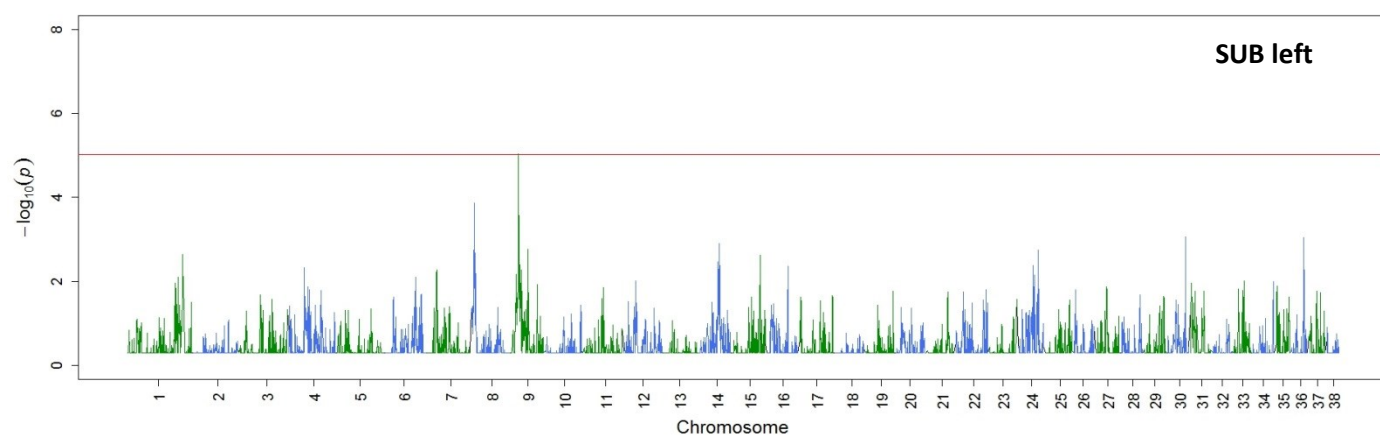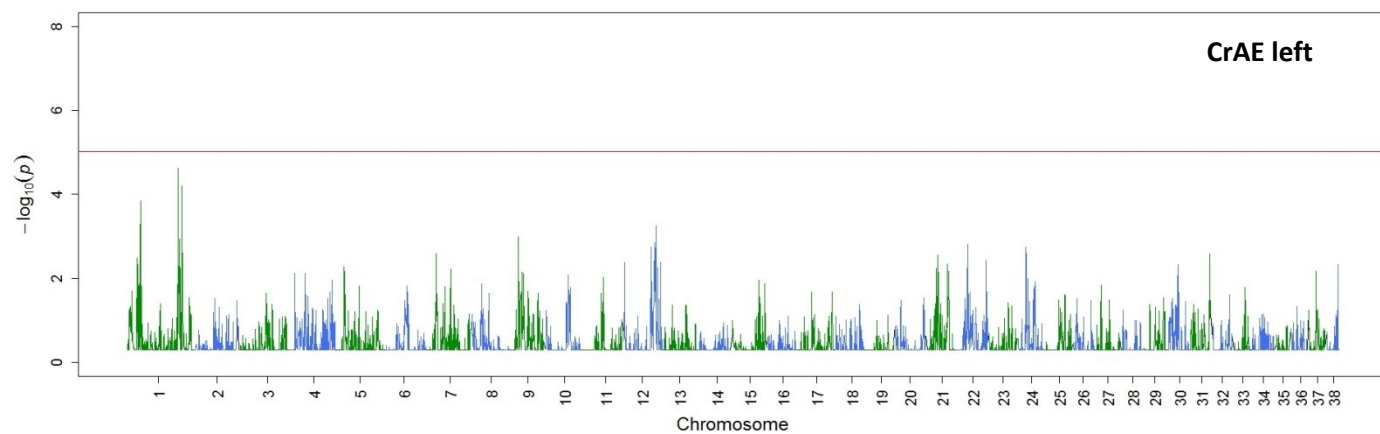

Supplement: Supplementary file 6 — Additional file 6: Regional heritability analysis. P-values for the significance of the genetic variance explained by each window of 20 SNPs for all traits, considering a Bonferroni-corrected threshold (red line). (PDF 1 MB) [file 12864_2014_6512_MOESM6_ESM.pdf]
